# Early molecular imaging response assessment based on determination of total viable tumor burden in [^68^Ga]Ga-PSMA-11 PET/CT independently predicts overall survival in [^177^Lu]Lu-PSMA-617 radioligand therapy

**DOI:** 10.1007/s00259-021-05594-8

**Published:** 2021-11-02

**Authors:** Florian Rosar, Felix Wenner, Fadi Khreish, Sebastian Dewes, Gudrun Wagenpfeil, Manuela A. Hoffmann, Mathias Schreckenberger, Mark Bartholomä, Samer Ezziddin

**Affiliations:** 1 Department of Nuclear Medicine, Saarland University – Medical Center, Kirrberger Str. 100, Geb. 50, 66421 Homburg, Germany; 2 Department of Biostatistics, Saarland University, Homburg, Germany; 3 Department of Nuclear Medicine, Johannes Gutenberg-University, Mainz, Germany

**Keywords:** Metastatic castration-resistant prostate cancer, Radioligand therapy, PSMA PET/CT, Molecular imaging, Response assessment

## Abstract

**Purpose:**

In patients with metastatic castration-resistant prostate cancer (mCRPC) treated with prostate-specific membrane antigen-targeted radioligand therapy (PSMA-RLT), the predictive value of PSMA PET/CT-derived response is still under investigation. Early molecular imaging response based on total viable tumor burden and its association with overall survival (OS) was explored in this study.

**Methods:**

Sixty-six mCRPC patients who received [^177^Lu]Lu-PSMA-617 RLT within a prospective patient registry (REALITY Study, NCT04833517) were analyzed. Patients received a [^68^Ga]Ga-PSMA-11 PET/CT scan before the first and after the second cycle of PSMA-RLT. Total lesion PSMA (TLP) was determined by semiautomatic whole-body tumor segmentation. Molecular imaging response was assessed by change in TLP and modified PERCIST criteria. Biochemical response was assessed using standard serum PSA and PCWG3 criteria. Both response assessment methods and additional baseline parameters were analyzed regarding their association with OS by univariate and multivariable analysis.

**Results:**

By molecular imaging, 40/66 (60.6%) patients showed partial remission (PR), 19/66 (28.7%) stable disease (SD), and 7/66 (10.6%) progressive disease (PD). Biochemical response assessment revealed PR in 34/66 (51.5%) patients, SD in 20/66 (30.3%), and PD in 12/66 (18.2%). Response assessments were concordant in 49/66 (74.3%) cases. On univariate analysis, both molecular and biochemical response (*p* = 0.001 and 0.008, respectively) as well as two baseline characteristics (ALP and ECOG) were each significantly associated with OS. The median OS of patients showing molecular PR was 24.6 versus 10.7 months in the remaining patients (with SD or PD). On multivariable analysis molecular imaging response remained an independent predictor of OS (*p* = 0.002), eliminating biochemical response as insignificant (*p* = 0.515).

**Conclusion:**

The new whole-body molecular imaging–derived biomarker, early change of total lesion PSMA (TLP), independently predicts overall survival in [^177^Lu]Lu-PSMA-617 RLT in mCRPC, outperforming conventional PSA-based response assessment. TLP might be considered a more distinguished and advanced biomarker for monitoring PSMA-RLT over commonly used serum PSA.

## Introduction

Prostate cancer (PC) is the second most common malignancy in men around the world and one of the leading causes for cancer-related mortality in elderly men [1]. While patients in early-PC stages generally have a good survival expectancy, some patients advance to a more aggressive and lethal stage of metastatic castration-resistant prostate cancer (mCRPC) with a poorer prognosis [2, 3]. Treatment options for patients presenting with mCRPC have evolved and improved in recent years. Ranging from taxane chemotherapy (docetaxel and cabazitaxel), novel androgen axis drugs (NAAD, e.g., abiraterone and enzalutamide), to bone-seeking radiotherapy with [^223^Ra]Ra-dichloride to PARP-inhibitors for patients with mutations in DNA repair genes [4–9]. If mCRPC progresses under these therapy options, radioligand therapy (RLT) targeting the prostate-specific membrane antigen (PSMA) is a promising alternative. PSMA is a transmembrane glycoprotein, which is overexpressed on the cell surface of prostate cancer cells offering new ways of imaging and treatment of PC [10–12]. PSMA-targeted radioligand therapy (PSMA-RLT) using ^177^Lu-labeled PSMA ligands as [^177^Lu]Lu-PSMA-617 has shown encouraging results in various retrospective studies [13–15], in prospective phase II trials [16, 17] and in a recently published phase III trial [18]. Response assessment to these treatments is routinely based on the biochemical parameter prostate-specific antigen (PSA) and conventional imaging modalities as computed tomography (CT), magnetic resonance tomography (MRI), and bone scintigraphy [19]. However, new parameters and imaging techniques are currently being investigated to assess response to treatment, especially in patients undergoing PSMA-RLT, as conventional imaging may be inappropriate in mCRPC [20, 21]. In recent years, PSMA-targeted PET/CT (using, e.g., [^68^Ga]Ga-PSMA-11) has gained increasing importance in the management of prostate cancer for initial staging, biochemical recurrence, and screening for PSMA-RLT [22–24]. The use of PSMA-targeted PET/CT for therapy monitoring and molecular imaging–based response assessment is currently the subject of ongoing research [25, 26]. Besides the PET-based assessment of individual target lesions, determination of total tumor burden by PET/CT might be a more suitable tool for response assessment [27, 28]. Following total lesion glycolysis (TLG), which is an established parameter for assessing total viable tumor burden on [^18^F]FDG-PET/CT [29], total lesion PSMA (TLP) may be a corresponding parameter for PSMA-targeted PET/CT [30]. However, the use of TLP in mCRPC, especially to monitor PSMA-RLT, still needs further investigation.

In this study, we investigated the value of early molecular imaging response assessment based on TLP for monitoring [^177^Lu]Lu-PSMA-617 RLT. TLP was obtained from [^68^Ga]Ga-PSMA-11 PET/CT and determined at baseline and after 2 cycles of [^177^Lu]Lu-PSMA-617 RLT. Molecular imaging and the established biochemical assessment of response were compared and evaluated as potential predictors of survival outcome.

## Methods

### Patient population and ethics

In this study, *n* = 66 patients with advanced mCRPC, who received [^177^Lu]Lu-PSMA-617 RLT in a palliative setting, were analyzed. Patients were treated at our institution within a prospective patient registry (REALITY Study, NCT04833517). Inclusion criteria for this study were confirmed mCRPC, at least 2 cycles of [^177^Lu]Lu-PSMA-617 RLT, [^68^Ga]Ga-PSMA-11 PET/CT before the first and after the second cycle of [^177^Lu]Lu-PSMA-617 RLT, absence of [^18^F]FDG/[^68^Ga]Ga-PSMA-11 mismatch findings (if additional [^18^F]FDG-PET/CT was performed), and availability of clinical outcome data. All patients received multiple therapies prior to PSMA-RLT, including ADT, NAAD, chemotherapy, and [^223^Ra]Ra-dichloride therapy. Detailed information about the patient characteristics is presented in Table 1. Between both PET scans, ADT and NAAD had to be continued unchanged to avoid altering PSMA expression [31]. PSMA-RLT was performed on a compassionate use basis under the German Pharmaceutical Act §13 (2b). Patients gave their consent after being thoroughly informed about the risks and potential adverse effects of PSMA-RLT. In addition, the patients agreed to the publication of the resulting data in accordance with the Declaration of Helsinki. The study was approved by the local Institutional Review Board (ethics committee permission number 140/17).

**Table 1:** Patient characteristics

Patient characteristics	Value
Age	
Median (range) Age ≥ 65 years, *n* (%)	71 (48–88) 49 (74.2)
PSA (ng/mL)	
Median (range)	145 (7–9579)
ALP (U/L)	
Median (range)	112 (35–1753)
Hemoglobin (g/dL)	
Median (range) < 13 g/dL, *n* (%)	12 (6–16) 44 (66.7)
ECOG performance status, *n* (%)	
0 1 ≥ 2	17 (25.8) 31 (47.0) 18 (27.3)
Sites of metastases, *n* (%)	
Bone Lymph node Liver Other	62 (93.9) 49 (74.2) 12 (18.2) 16 (24.2)
Prior therapies, *n* (%)	
Prostatectomy Radiation ADT NAAD Abiraterone Enzalutamide Abiraterone and enzalutamide Chemotherapy Docetaxel Cabazitaxel Docetaxel and cabazitaxel [^223^Ra]Ra-dichloride Other	32 (48.5) 41 (62.1) 66 (100) 65 (98.5) 50 (75.8) 54 (81.8) 39 (59.1) 47 (71.2) 46 (69.7) 21 (31.8) 20 (30.3) 14 (21.2) 11 (16.7)

*PSA*, prostate-specific antigen; *ALP*, alkaline phosphatase; *ECOG*, Eastern Cooperative Oncology Group; *ADT*, androgen deprivation therapy; *NAAD*, novel androgen axis drugs

### [^177^Lu]Lu-PSMA-617 RLT

All patients received two cycles of [^177^Lu]Lu-PSMA-617 RLT. The mean interval between the two cycles was 5 ± 2 weeks. [^177^Lu]Lu-PSMA-617 was synthesized according to previously published standard procedures [32]. PSMA-617 was obtained from ABX advanced biochemical compounds GmbH (Radeberg, Germany) and ^177^Lu from IDB Holland BV (Baarle-Nassau, The Netherlands). For labeling, 150 μg (143 nmol) PSMA-617 were used for 6 GBq of ^177^Lu. Radiochemical yields and purity of the radiotracer were ≥ 99%. The administered activities were individually adjusted to patient’s specific characteristics such as body surface, tumor progression dynamics, distribution and extent of tumor burden, bone marrow, and renal function. The median applied activity per cycle was 7.1 GBq (range: 4.3–11.6 GBq). The median administered activity was slightly higher in the first cycle compared to that in the second cycle (median 7.2 versus 6.7 GBq, *p* < 0.001). The median cumulative activity after the 2 cycles of [^177^Lu]Lu-PSMA-617 was 14.1 GBq (range: 9.0–19.4 GBq). Each patient received intravenous hydration (500 mL 0.9% NaCl) and cooling of the salivary glands, starting 30 min prior to treatment infusion. The [^177^Lu]Lu-PSMA-617 solution was administered intravenously by infusion line over a period of 1 h. No diuretics or other renal protection was applied.

### [^68^Ga]Ga-PSMA-11 PET/CT

Each patient received a [^68^Ga]Ga-PSMA-11 PET/CT 2 ± 2 weeks before the first and 5 ± 2 weeks after the second cycle of [^177^Lu]Lu-PSMA-617 RLT. PSMA-11 was obtained from ABX advanced biochemical compounds GmbH (Radeberg, Germany) and ^68^Ga using an ^68^Ge/^68^Ga generator provided by Eckert & Ziegler Strahlen- und Medizintechnik AG (Berlin, Germany). Administration of median 125 MBq (range 77–166 MBq) [^68^Ga]Ga-PSMA-11 was performed intravenously followed by a 500 mL infusion of 0.9% NaCl. Applied activities did not differ significantly (*p* = 0.192) between the two PET/CT scans. No additional diuretics were given. Before infusing the tracer, blood samples were taken and tested for routine laboratory parameters including PSA, alkaline phosphatase (ALP), and full blood count. The time from injection to the PET acquisition was approximately 60 min according to standard procedures for prostate cancer imaging [33]. PET/CT scans were performed on a Biograph 40 mCT PET/CT scanner (Siemens Medical Solutions, Knoxville, TN, USA) (acquisition time: 3 min/bed position; extended field of view: 21.4 cm (TrueV); slice thickness: 3.00 mm) with EANM Research Ltd. accreditation. A low-dose CT was acquired for attenuation correction and anatomical localization using an x-ray tube voltage of 120 keV and a modulation of the tube current applying CARE Dose4D with a reference tube current of 50 mAs. CT scans were reconstructed as 512 × 512 matrix with an increment of 3.0 mm and a slice thickness of 5.00 mm. PET reconstruction was performed iteratively utilizing a three-dimensional OSEM (ordered-subset expectation maximization) algorithm with three iterations, 24 subsets, Gaussian filtering, and a slice thickness of 5.00 mm. Decay correction, random correction, scatter correction, and attenuation correction were implemented.

### Response assessment

**Fig. 1:**
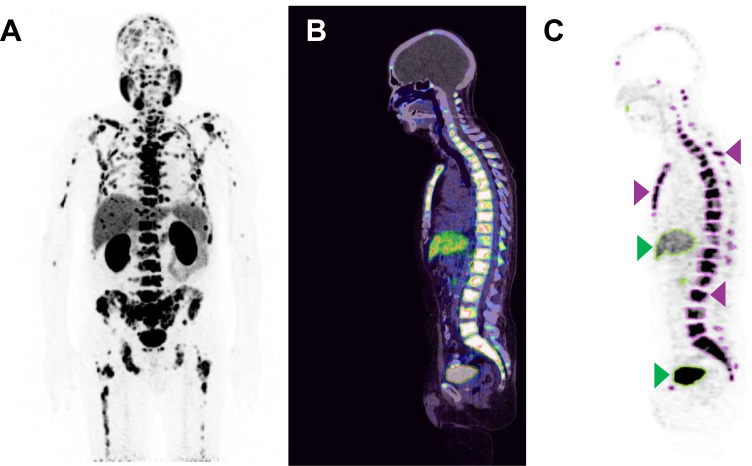
Example of tumor delineation using Syngo.Via. **A** Maximum intensity projection of [^68^Ga]Ga-PSMA-11 PET/CT. **B** PET/CT fusion (sagittal plane). **C** Tumor delineation in a sagittal PET slice with semiautomatically drawn volumes of interest (VOI). Tumor lesions are bordered violet (arrows point to exemplary bone lesions). Physiological uptake sites with green outline (arrows point to the liver and bladder) were manually excluded

The pre- and post-therapy [^68^Ga]Ga-PSMA-11 PET/CT scans were analyzed applying a semiautomatic tumor segmentation algorithm using Syngo.Via (Enterprise VB 40B, Siemens, Erlangen, Germany) with a threshold of standardized uptake value (SUV) ≥ 3 as previously described by Ferdinandus et al. [34]. Physiologic [^68^Ga]Ga-PSMA-11 uptake sites such as the salivary glands, vocal cords, liver, spleen, intestine, ureter, and the bladder were manually excluded if these presented with an SUV above the threshold. For the segmentation of liver metastases, a threshold of 1.5 × SUV_mean_ of the healthy liver tissue was used. Total lesion PSMA (TLP), defined as the summed products of volume × uptake (SUV_mean_) of all lesions, was calculated. Figure 1 illustrates the process of tumor delineation using Syngo.Via.

For molecular imaging response assessment based on TLP, we followed thresholding as used in PERCIST 1.0 criteria [35] to determine partial remission (PR), stable disease (SD) and progressive disease (PD). PR was defined as a TLP decline > 30%, PD as an increase > 30%, and SD as a change between − 30 and + 30%.

For biochemical response assessment, we applied the Prostate Cancer Working Group 3 (PCWG3) criteria and defined PD as a PSA increase of > 25% [19]. PR was defined as a PSA decline of > 50% and SD as a change between − 50 and + 25%. PSA serum values were measured on the same days when the PET scans were performed.

### Statistical analysis

For statistical analysis, SPSS version 27 (IBM Corp., Armonk, USA) and Prism version 8 (GraphPad Software, San Diego, USA) were used. Besides descriptive and correlation analyses (using Spearman’s rank correlation test), survival analyses were performed. A *p*-value of < 0.05 was regarded as statistically significant. Overall survival (OS) was defined as the interval from the start of PSMA-RLT to the time point of (1) death from any cause or (2) the last study visit. The cutoff follow-up date was June 30, 2021. Median follow-up and OS were analyzed using the Kaplan–Meier method. Patients were independently dichotomized by molecular imaging and biochemical response assessments into two groups: (a) patients with PR and (b) patients with SD or PD. In addition, patients were categorized by presence of visceral metastases, age, ECOG performance status, hemoglobin level, ALP level, viable tumor burden measured by TLP, PSA level at the start of [^177^Lu]Lu-PSMA-617 RLT, and cumulative ^177^Lu activity after 2 cycles, using respective cutoffs of 65 years, ECOG 2, 13 g/dL, 220 U/L, 5710 mL × SUV, 145 ng/mL, and 14 GBq. For each variable, univariate regression was performed. Variables contributing to the univariate model (*p* < 0.1) were included in multivariable analysis using a stepwise model by backward elimination to identify independent predictors for OS.

## Results

### Molecular imaging and biochemical response

**Fig. 2:**
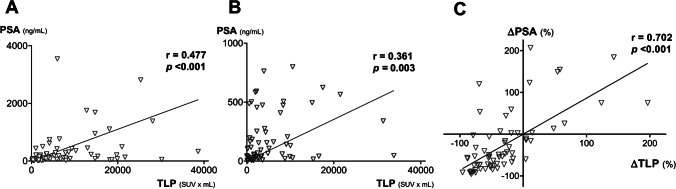
Correlation between **A** baseline PSA and TLP, **B** PSA and TLP after 2 cycles of PSMA-RLT, and **C** ∆TLP and ∆PSA. One outlier in **A** with PSA > 4000 ng/mL was cropped for clearness

At baseline, patients had a median TLP and PSA of 5710 mL × SUV (range: 130–38,638 mL × SUV) and 145 ng/mL (range: 7–9579 ng/mL), respectively. After 2 cycles of [^177^Lu]Lu-PSMA-617 RLT, median TLP and PSA were 2610 mL × SUV (range: 40–33,793 mL × SUV) and 67 ng/mL (range: 1–799 ng/mL), respectively. Median ∆TLP and ∆PSA were − 44% (range: − 96–197%) and − 53% (range: − 96–207%), respectively. Correlation analyses (Fig. 2) revealed a significantly moderate correlation between baseline PSA and TLP (*r* = 0.477, *p* < 0.001), a significantly low correlation between post-treatment PSA and TLP (*r* = 0.361, *p* = 0.003), and a significantly strong correlation between ∆PSA and ∆TLP (*r* = 0.702, *p* < 0.001).

**Fig. 3:**
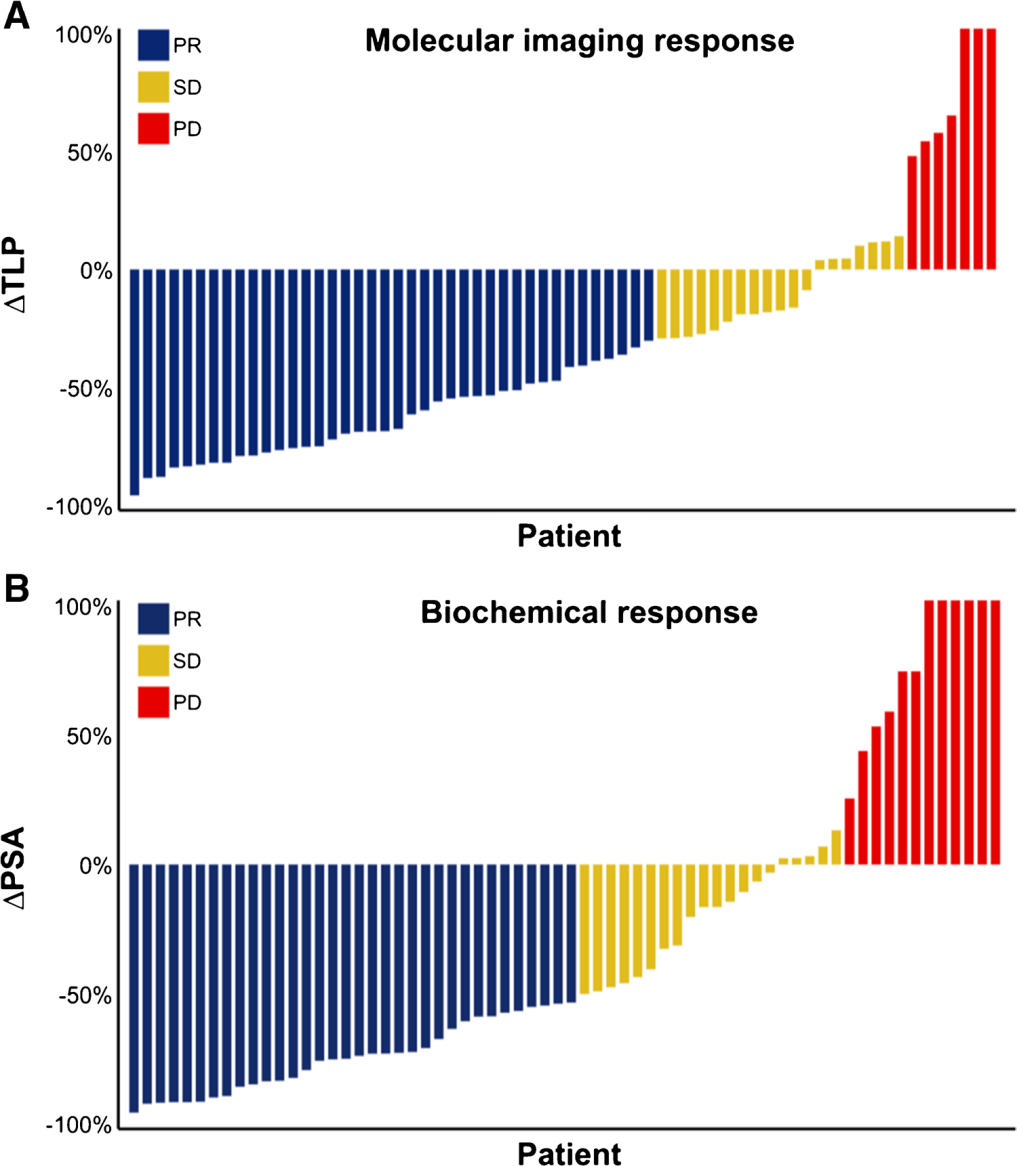
Waterfall plots visualizing relative changes in **A** TLP and **B** PSA, concurrently illustrating molecular imaging and biochemical response assessment. PR = partial remission, SD = stable disease, PD = progressive disease. Values over 100% were cropped for simplicity

Using molecular imaging response assessment based on change of TLP, 40 patients (60.6%) were classified as PR, 19 patients (28.7%) as SD, and 7 patients (10.6%) as PD. Biochemical response assessment by PSA revealed PR in 34 patients (51.5%), SD in 20 patients (30.3%), and PD in 12 patients (18.2%). Individual changes in ∆TLP and ∆PSA along with corresponding response assessment are presented in Fig. 3.

**Fig. 4:**
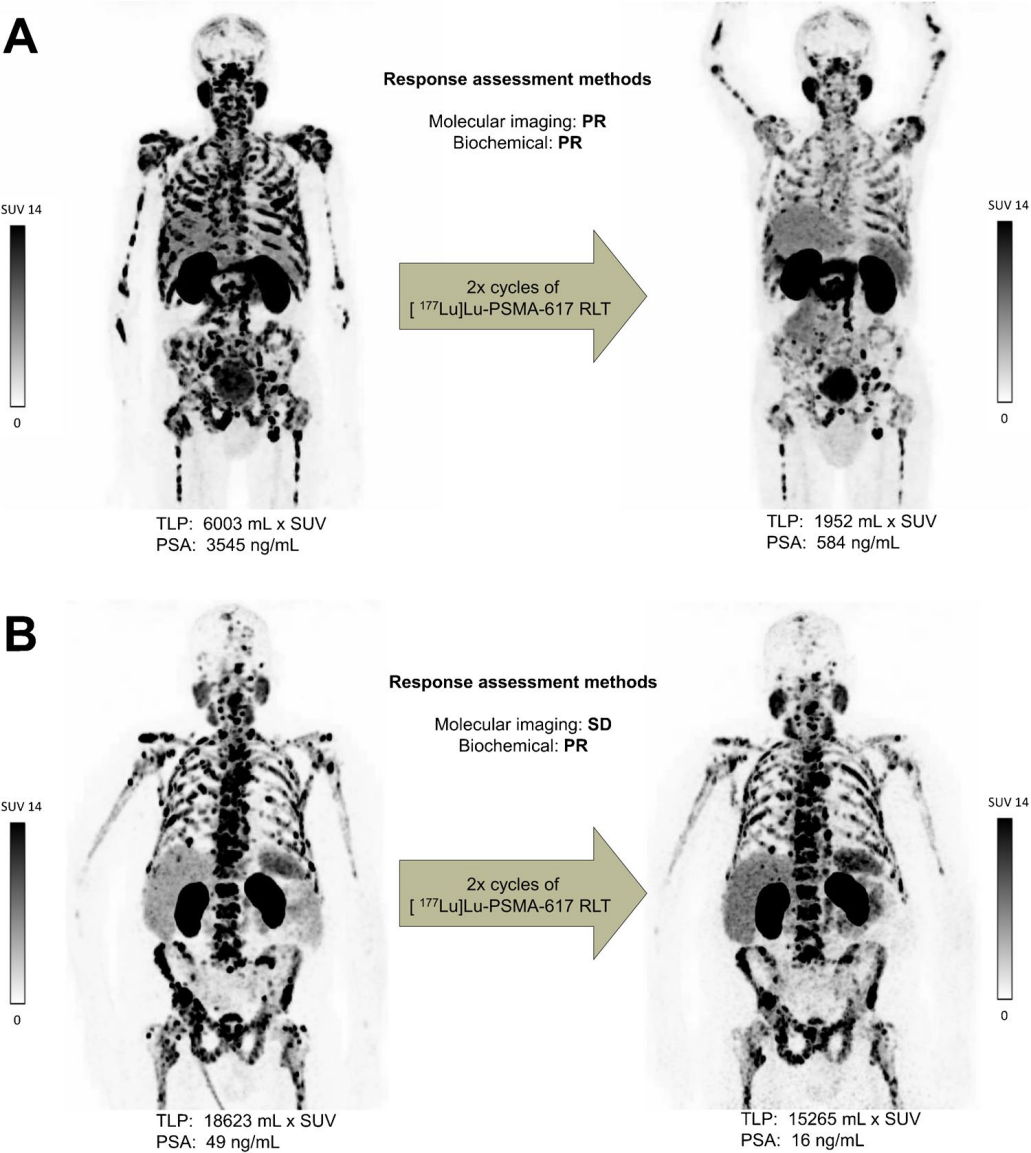
Examples of concordance and discordance between molecular imaging and biochemical response assessment. **A** Patient no. 35: classified as partial remission (PR) by both assessment methods (∆TLP: − 67%; ∆PSA: − 84%). **B** Patient no. 31: classified as stable disease (SD) by molecular imaging (∆TLP: − 18%) and as PR by biochemical response assessment (∆PSA: − 67%)

Concordance of biochemical and molecular imaging response assessment was found in 49/66 patients (74.3%). Seventeen patients (25.7%) revealed discordance between both assessment methods. With the exception of three patients, all patients who showed PR by PSA also showed PR by molecular imaging. These patients were classified by molecular imaging as having SD. Eight cases of discordance were found in patients with SD by PSA. Seven of them revealed PR and one PD by molecular imaging. Six patients with PD by PSA revealed discrepant molecular imaging responses (four SD and two PR). Figure 4 depicts each as an example of concordant and of discordant molecular imaging versus biochemical response assessment.

### Survival analysis

After the completion of the 2 cycles [^177^Lu]Lu-PSMA-617 RLT, 63/66 (95.5%) patients continued PSMA-RLT with a median of 4 cycles (range: 1–16 cycles). Due to progression in the further course, 24 patients received [^225^Ac]Ac-PSMA-617 augmented [^177^Lu]Lu-PSMA-617 RLT after median 5 cycles (range 2–9 cycles) and 6 patients received chemotherapy after median 5 cycles (range 2–8 cycles). The median follow-up time was 23.5 months (95% confidence interval (CI): 16.9–30.1 months). By the end of the study, 41/66 patients (62.1%) died. All deaths were mCRPC-related. No treatment-related death was observed. The median OS was 18.0 months (95% CI: 14.6–21.4 months).

**Fig. 5:**
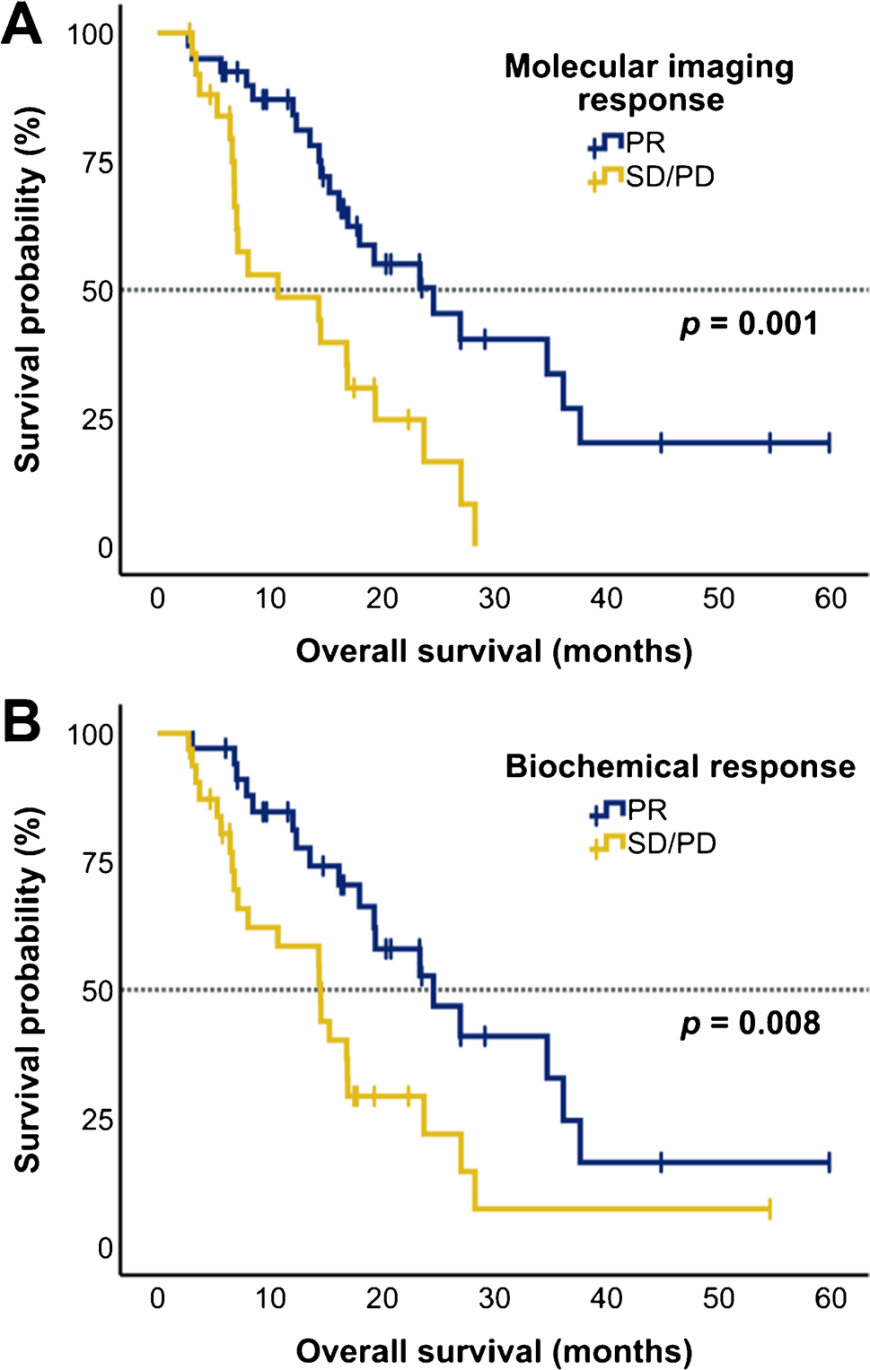
Kaplan–Meier plots illustrating overall survival (OS) stratified by **A** molecular imaging response based on TLP and **B** biochemical response determined by ∆PSA. PR = partial remission, SD = stable disease, PD = progressive disease

In the univariate analysis, both response assessments and two baseline characteristics (ALP and ECOG) were significantly associated with OS (Table 2). Patients showing PR by molecular imaging response assessment after 2 cycles of [^177^Lu]Lu-PSMA-617 RLT had significantly (*p* = 0.001, log-rank test) longer OS than patients classified with SD or PD. The median OS was 24.6 months (95% CI 15.4–33.8 months) and 10.7 months (95% CI 0–21.8 months), respectively. Patients showing biochemical PR had also significantly longer OS than patients with biochemical SD or PD with a median OS of 24.6 months (95% CI 15.5–33.7 months) versus 14.5 months (9.6–19.4 months, *p* = 0.008). The corresponding Kaplan–Meier curves are shown in Fig. 5.

**Table 2:** Univariate and multivariable analysis of potential predictive factors for overall survival (OS)

Variable	*n* (%)	OS (months)	Univariate analysis		Multivariable analysis	
		Median (95% CI)	HR (95% CI)	*p*	HR (95% CI)	*p*
Overall	66 (100)	18.0 (14.6–21.4)	-	-	-	-
TLP response						
PR	40 (60.6)	24.6 (15.4–33.8)				
SD/PD	26 (39.4)	10.7 (0–21.8)	2.78 (1.46–5.30)	**0.001**	2.76 (1.45–5.26)	**0.002**
PSA response						
PR	34 (51.5)	24.6 (15.5–33.7)				
SD/PD	32 (48.5)	14.5 (9.6–19.4)	2.30 (1.23–4.30)	**0.008**	1.39 (0.52–3.69)	0.515
ALP^a^						
< 220 U/L	54 (81.8)	23.4 (16.4–30.4)				
≥ 220 U/L	12 (18.2)	7.1 (0–15.5)	4.08 (1.90–8.76)	** < 0.001**	3.08 (1.38–6.87)	**0.006**
Performance status^a^						
ECOG < 2	48 (72.7)	23.4 (17.1–29.6)				
ECOG ≥ 2	18 (27.3)	8.1 (0–17.0)	2.98 (1.53–5.79)	** < 0.001**	2.21 (1.10–4.43)	**0.026**
Visceral metastases^a^						
No	43 (65.2)	19.3 (8.7–29.9)				
Yes	23 (34.8)	16.2 (8.9–23.4)	1.78 (0.96–3.32)	0.064	1.61 (0.84–3.08)	0.154
PSA^a^						
< 145 ng/mL	33 (50.0)	23.4 (15.6–31.1)				
≥ 145 ng/mL	33 (50.0)	16.2 (12.2–20.1)	-	0.105	-	-
Age^a^						
< 65 years	17 (25.8)	16.8 (11.1–22.6)				
≥ 65 years	49 (74.2)	19.3 (15.1–23.5)	-	0.473	-	-
Prior chemotherapy^a^						
No	19 (28.8)	23.7 (12.0–35.4)				
Yes	47 (71.2)	16.9 (11.5–22.3)	-	0.570	-	-
Hemoglobin^a^						
≥ 13 g/dL	22 (33.3)	18.0 (12.5–23.5)				
< 13 g/dL	44 (66.7)	16.9 (10.8–23.0)	-	0.566	-	-
TLP^a^						
< 5710 mL × SUV	33 (50.0)	19.4 (11.1–27.8)				
≥ 5710 mL × SUV	33 (50.0)	16.9 (10.3–23.5)	-	0.312	-	-
Initial ^177^Lu activity^b^						
> 14 GBq	33 (50.0)	16.8 (14.4–19.3)				
≤ 14 GBq	33 (50.0)	23.7 (11.5–35.9)	-	0.474	-	-

*CI*, confidence interval; *HR*, hazard ratio; *TLP*, total lesion PSMA; *PSA*, prostate-specific antigen; *ALP*, alkaline phosphatase; *ECOG*, Eastern Cooperative Oncology Group. ^a^Baseline parameter. ^b^Cumulative activity of the first two [^177^Lu]Lu-PSMA-617 RLT cycles. *p*-values printed in bold type are statistically significant at *p* < 0.05

In the multivariable analysis, the molecular imaging response assessment remained an independent predictor of OS with a hazard ratio (HR) of 2.76 (*p* = 0.002) for patients classified as PD/SD, relative to patients with PR. High ALP levels ≥ 220 U/L and an ECOG ≥ 2 also remained independently predicting OS with an HR of 3.08 (*p* = 0.006) and 2.21 (*p* = 0.026), respectively (Table 2). Biochemical response did not remain significant in multivariable analysis (*p* = 0.515).

## Discussion

The aim of this study was to evaluate whole-body molecular imaging response assessment for [^177^Lu]Lu-PSMA-617 RLT, based on the determination of total viable tumor burden in [^68^Ga]Ga-PSMA-11 PET/CT. Total viable tumor burden was derived by calculating TLP, a parameter considering both volume and PSMA density of all metastases. The results of this study in *n* = 66 mCRPC patients demonstrates that early molecular imaging response assessment using TLP independently predicts OS.

After 2 cycles of PSMA-RLT, 60.6% (40/66) of the patients showed PR; only 28.8% (19/66 patients) and 10.6% (7/66) showed SD or PD based on molecular imaging. There are only a few studies on molecular imaging–based response assessment after PSMA-RLT and all differ in methodology [25–28]. Grubmüller el al. based the molecular imaging response assessment (in *n* = 38 patients) on change in whole-body tumor volume and reported a response rate of 63% [28]. Whereas Khreish et al. and Kurth et al. based the assessment (in *n* = 51 patients and *n* = 29) on change of PSMA expression in target lesions and reported response rates after 2 cycles PSMA-RLT of 69% and 29% [25, 26]. Analogous to our methodology, the combination of uptake and volume represented by TLP as a parameter for response assessment was previously reported by Michalski et al. in a small cohort of patients (*n* = 10) [27]. The authors reported a decrease of TLP > 30% in 60% of patients, which is in line with our results.

PSA and TLP values showed only moderate correlation (*r* = 0.477) at baseline, and even poorer correlation (*r* = 0.361) after 2 cycles PSMA-RLT. Between ∆PSA and ∆TLP; however, we found a strong correlation (*r* = 0.702). Concordance analysis between molecular imaging and biochemical response assessment revealed a concordance of 74.3% (49/66) between both methods in our study. Comparable concordances of 63–87% have been reported in other studies [27, 28, 36].

To the best of our knowledge, this is the first study showing that molecular imaging response assessment based on TLP is strongly and independently associated with OS. Patients with PR showed a significantly longer median OS than patients with SD or PD (24.6 versus 10.7 months, *p* = 0.001). Multivariable analysis identified the strong association with OS as independent from and superior over the change in PSA, underlining the powerful predictive value of TLP-based response assessment. Grubmueller et al. and Kurth et al. also showed that molecular imaging response assessment based on whole-body tumor volume or target lesions after 2 cycles of PSMA-RLT can predict OS; however, both did not perform multivariable analysis [26, 28]. Since in our study on biochemical—in contrast to molecular imaging—response assessment was only associated with survival on univariate analysis and did not remain an independent predictor of OS on multivariable analysis, we conclude its redundant and inferior predictive information compared to molecular imaging–based response assessment. Based on our results, TLP is a suitable parameter for response assessment in analogy to TLG in [^18^F]FDG-PET/CT for response assessments in other tumor entities [37–39]. Further studies in larger cohorts, ideally in prospective settings, would be warranted to confirm our results.

Despite this arguable superiority of molecular imaging response assessment using TLP over the established biochemical response assessment, it must be noted that calculating TLP is a time-consuming procedure making implementation in clinical practice challenging. Furthermore, it must be pointed out that there are several methods for calculating TLP. Even though percentage-based thresholding, e.g., 41% or 50% of SUV_max_, is recommended by the European Association of Nuclear Medicine for assessing TLG in [^18^F]FDG-PET/CT [29], we decided to apply the method published by Ferdinandus et al. [34] with a fixed SUV threshold of 3.0 to avoid underestimating lesion volume in case of heterogeneous PSMA expression, which is often present in disseminated and confluent disease after therapy. For delineating liver metastases, we used a threshold of 1.5 × SUV_mean_ of the healthy liver, which appeared to be appropriate compared to visual findings. Further studies in this field are needed to evaluate which criteria and settings for semiautomatic tumor segmentation are the most suitable for [^68^Ga]Ga-PSMA-11 PET/CT to determine whole-body total tumor load. An intriguing application that could facilitate the process of tumor segmentation and thus enable broader clinical applicability in near future is the use of artificial intelligence (AI) to determine tumor burden with greater speed and ease. The feasibility of segmentation employing AI in determining tumor burden has recently been demonstrated for [^18^F]FDG-PET/CT scans in patients with lung cancer and lymphoma [40–42]. However, data on applying AI-based segmentation in [^68^Ga]Ga-PSMA-11 PET/CT for patients with mCRPC is still lacking.

Another interesting approach for PSMA-PET-based response assessment in metastatic prostate cancer, the PSMA PET Progression (PPP) criteria, was proposed by Fanti et al., where imaging data (number and location of newly appeared metastases, increase in uptake or size) is complemented by biochemical and clinical parameters [43]. While total tumor burden is not included in this approach, an integration of our biomarker TLP into PPP criteria might also be worth further investigation, especially in advanced mCRPC.

We found two baseline parameters, serum ALP level and ECOG performance status, that were also independently predictive of OS in our study, which is in accordance with various previously published studies on PSMA-RLT [15, 44, 45] and other treatments of mCRPC [46–48].

The results reported herein should be considered in the light of some limitations. First of all, this single-center study suffers from the somewhat limited number of patients, although the series may present one of the largest molecular imaging response assessment studies. A second limitation may be seen in the performance of CT only with non-contrast-enhanced low-dose technique, lacking the option of response assessment according to Response Evaluation Criteria in Solid Tumors (RECIST). Molecular imaging response was assessed only after the second cycle of PSMA-RLT in this study; however, assessment after the first cycle would also be worthy of evaluation. It should also be noted that the observed median OS was longer than in other retrospective and prospective studies on PSMA-RLT, probably due to a selection bias by including only patients with at least 2 cycles of PSMA-RLT and the exclusion of patients with [^18^F]FDG/[^68^Ga]Ga-PSMA-11 mismatch findings (*n* = 5), known to be associated with worse prognosis [49]. In addition, about one-third of the patients received an additional augmentation of PSMA-RLT by [^225^Ac]Ac-PSMA-617 as a tandem therapy approach in the further course of disease, which may prolong survival [50, 51] and thereby impact survival analyses.

## Conclusion

[^68^Ga]Ga-PSMA-11 PET/CT-derived molecular imaging response assessment based on the change of whole-body total lesion PSMA (TLP) independently predicts overall survival in [^177^Lu]Lu-PSMA-617 RLT in mCRPC, outperforming conventional PSA-based response assessment. TLP can therefore be considered a more distinguished and advanced biomarker for monitoring PSMA-RLT over commonly used serum PSA. Larger studies, ideally in prospective settings, would be justified to confirm this initial evidence.

## Data Availability

The datasets used and analyzed during the current study are available from the corresponding author on reasonable request.

## Abbreviations

ADT: Androgen deprivation therapy
AI: Artificial intelligence
ALP: Alkaline phosphatase
CT: Computed tomography
EANM: European Association of Nuclear Medicine
ECOG: Eastern Cooperative Oncology Group
FDG: Fluordesoxyglucose
mCRPC: Metastatic castration-resistant prostate cancer
MRI: Magnetic resonance imaging
NAAD: Novel androgen axis drugs
OS: Overall survival
PARP: Poly adenosine diphosphate-ribose polymerase
PC: Prostate cancer
PCGW3: Prostate Cancer Working Group 3
PD: Progressive disease
PERCIST: Positron Emission Response Criteria in Solid Tumors
PET: Positron emission tomography
PPP: PSMA PET progression
PR: Partial remission
PSA: Prostate-specific antigen
PSMA: Prostate-specific membrane antigen
PSMA-RLT: PSMA-targeted radioligand therapy
RECIST: Response Evaluation Criteria in Solid Tumors
RLT: Radioligand therapy
SD: Stable disease
SUV: Standardized uptake value
TLG: Total lesion glycolysis
TLP: Total lesion PSMA
